# Why Human Health and Health Ethics Must Be Central to Climate Change Deliberations

**DOI:** 10.1371/journal.pmed.1001229

**Published:** 2012-06-05

**Authors:** Jerome Amir Singh

**Affiliations:** 1 Centre for the AIDS Programme of Research in South Africa (CAPRISA), University of Kwazulu-Natal, Durban, South Africa; 2 Dalla Lana School of Public Health, Joint Centre for Bioethics, and Sandra Rotman Centre, University of Toronto, Toronto, Canada

## Abstract

Jerome Singh argues that health ethics principles must be afforded equal status to economics principles in climate change deliberations, and that the health community must play more of a leadership role.

**Summary Points**

- The human health implications of climate change must be afforded greater prominence.
- Governments, the private sector, financiers, and society have a moral responsibility to practice socially responsible investment and to mitigate against the impact of climate change, particularly in relation to human health.
- Human health must be a core, not peripheral, focus in future climate change deliberations.
- The health community, led by health ministers, must play a central role in climate change deliberations.
- Health ethics principles must be afforded equal status to economics principles in climate change deliberations.

## Human Health and Climate Change

The 17th Conference of the Parties to the United Nations Convention on Climate Change (COP 17) concluded in December 2011, in Durban, South Africa, two days late, after two weeks of negotiations. What ultimately emerged was a further voluntary commitment period for the Kyoto Protocol international instrument that sets binding targets for 37 industrialized countries and the European community for reducing greenhouse gas (GHG) emissions, and expires in 2012—and more significantly, the Durban Platform for Enhanced Action. This is an agreement that commits governments to developing a protocol, legal instrument, or an agreed outcome to cut greenhouse gas (GHG) emissions with legal force applicable to all countries by no later than 2015, under the United Nations Framework Convention on Climate Change (UNFCCC), to be implemented from 2020 [1].

Some regard the outcome of the Durban meeting to be a failure because it will not result in action fast enough [2],[3]. Scientists largely concur that if global temperatures rise 2°C beyond pre-industrial levels, this will have extremely negative and irreversible planetary consequences [4],[5]. To remain within this ceiling, the current rising trend in global emissions will have to be stemmed by 2020 [6]. This will not happen if a binding agreement only takes effect in 2020, especially as the World Meteorological Organization announced at the start of COP 17 that concentrations of greenhouse gases in the atmosphere reached new highs in 2011 and are very rapidly approaching levels consistent with a 2–2.4°C rise in average global temperatures [7],[8]. Others, such as the United Nations, hail COP 17 a breakthrough as the world's biggest polluters, including China, the United States, and India, accepted for the first time that an international treaty on climate change should bind them too.

Regardless of COP 17's perceived failure or success, unequivocally clear was that foreign ministers and environmental ministers set and drove the conference agenda, and that economic considerations underpinned all discussions. Despite climate change posing grave risks to human health (see Box 1), and the World Health Organization (WHO) prospectively devising a policy proposal for COP 17 [9], the human health perspective on climate change was relegated to side-event status at COP 17 [10]. This is also despite COP 17 hosting the largest health presence in a UNFCCC conference to date [11], and spawning a parallel inaugural Global Climate and Health Summit, which yielded the Durban Declaration on Climate and Health and Health Sector Call to Action [12]. The marginalisation of human health considerations at UNFCCC conferences is untenable. Human health must be a core, not peripheral, focus at future COP meetings [13]. Furthermore, the WHO and health ministers should help set and drive UNFCCC conference agendas. Similarly, health ethics considerations should be given equal weighting to economic considerations at future UNFCCC conference negotiations.

**Box 1. The Impact of Climate Change on Human Health**

The World Health Organization (WHO), the World Meteorological Organization, and the United Nations Environmental Programme have noted, with concern, the implications of climate change on human health.

The United States Interagency Working Group on Climate Change and Health has identified at least eleven categories of climate change's impact on human health, including:

asthma and respiratory disease;
cancer;
cardiovascular disease and stroke;
foodborne diseases and nutrition;
human developmental effects;
mental health and stress-related disorders;
neurological diseases;
vectorborne and zoonotic diseases;
waterborne diseases;
weather-related morbidity and mortality.

The Wildlife Conservation Society has identified 12 pathogens—dubbed the “deadly dozen”—that could spread into new regions and affect human health as a result of climate change:

avian flu;
tuberculosis;
Ebola virus;
cholera;
babesiosis;
parasites;
Lyme disease;
plague;
Rift Valley fever;
sleeping sickness;
yellow fever;
red tides (algal blooms).

Key references: [9],[28]–[38].

## Why Ethics Is Crucial to Climate Change Deliberations

While movement towards a legally binding international treaty on climate change is welcomed, and while many countries are moving towards domestic legal frameworks to govern greenhouse gas emissions in their settings, these governance instruments could be silent on issues such as the duty of care and custodianship towards resources and the environment that current generations owe future generations (intergenerational justice), or their adherence may not necessarily yield ethical outcomes. For example, COP 17's host, South Africa, is currently experiencing an energy security crisis, with the country's electricity grid network under strain [14]. In response, the country's state energy company, Eskom, with endorsement from the South African government [15], is building the Medupi and Kusile coal-fired power stations, and has successfully sought multi-billion funding for these power stations from the World Bank [16] and the US government's Export-Import Bank [17]. This is ethically questionable given that coal-powered stations are the biggest contributors to global warming. For example, US coal-fired power plants released 72% of US greenhouse gases reported for 2010 to the US Environmental Protection Agency [18]. Alarmingly, an estimated 6,000 and 10,700 annual deaths just from cardio-pulmonary diseases and cancer can be attributed to the 88 coal-fired power plants and companies that received public international financing, including from the World Bank [19],[20]. Further, more than US$37billion in direct funding and over US$100 billion in indirect financial support for new coal-fired generation has been provided by multilateral development banks and export credit agenciessince 1994, compared to the $6.36 billion mobilized by the United Nations Global Environment Facility for climate change mitigation over the same period [21].

World Bank funding for coal-powered stations comes despite the World Bank recognizing the impact of development on the environment [22]–[24]. This anomaly highlights that the World Bank and other international public financial institutions are not adequately practicing socially responsible investment, and have not given due regard to environmental, social, governance (ESG) factors in relation to some of the projects in which they are investing. Human health implications and ethics should be central to their feasibility and risk-benefit evaluation process. For example, if the public health ethics principle of harm prevention were to be applied to the issue of energy security in South Africa, it would hold that if the existing or proposed project or policy (coal-source energy) is impacting (or could impact) climate change and human health, and could be realised by feasible alternatives that are less harmful to human health (such as solar- or wind-based power), the latter alternatives should be pursued as a first resort. Development banks should practice the principle of beneficence by dedicating increased support for research and development of clean technologies, as this will ultimately improve the effectiveness and feasibility of these technologies. Conversely, financiers should practice the principle of non-maleficence and refrain from sponsoring developments (such as coal-powered plants) that will likely detrimentally impact human health.

The gap in ethics governance concerning climate change decision-making underscores the argument that policy-making on a variety of issues impacting climate change, including energy, transport, and development, needs to be underpinned by ethically sound principles, not just economic and legal considerations. Further, the human health implications of energy, transport, and development must be given equal weighting in policy deliberations as economic implications. While the field of climate justice has largely focused on distribution, development, and justice, particularly in relation to intergenerational justice and reparations for vulnerable states that stand to be affected by climate change [25], the field of health ethics should also become a central component of future risk management in relation to climate change.

In December 2004, the Collaborative Program on the Ethical Dimensions of Climate Change was launched at the 10th Conference of Parties to the United Nations Framework Convention on Climate Change (COP 10) in Buenos Aires, Argentina. The major outcome of this meeting was the Buenos Aires Declaration on the Ethical Dimensions of Climate Change [26]. While the Declaration was an important first step in recognising the role of ethics in climate change deliberations, it does not contain a guidance evaluative framework, and did not integrate principles of ethics from a variety of fields. The Declaration also raises general questions for consideration, rather than suggesting concrete ethics guidance or governance principles. For instance, the Declaration poses the following questions without answering them: “What ethical principles should guide the choice of specific climate change policy objectives including but not limited to maximum human-induced warming, and atmospheric greenhouse gas targets? What ethical principles should be followed in allocating responsibility among people, organizations, and governments at all levels to prevent ethically intolerable impacts from climate change?” As a result, the Declaration has failed to serve as an adequate guidance framework to national and international policy-makers or climate change negotiators.

Addressing the questions the Declaration poses will require more than just drawing on principles from the fields of environmental ethics, economic ethics, and climate justice. While the ethical dimensions of climate change have received recent attention [27], to date health ethics has been absent in climate change discourse. Going forward, any ethics-based climate change guidance framework for investors, policy-makers, and the private sector should incorporate relevant principles from the fields of bioethics, public health ethics, and global health ethics (see Box 2 for a proposed set of principles drawn from the field of health ethics). These should be integrated in addition to environmental ethics, climate justice (reciprocal justice and intergenerational justice), economics ethics, and socially responsible corporate governance. Given that deliberations for a binding international treaty commence at COP 18 in Doha 2012, there is an urgent need to devise such a multi-disciplinary synergized framework so that it can influence deliberations at future COP meetings, especially in the run-up to 2015, when a legally binding agreement is expected to come into effect, as outlined in the Durban platform.

**Box 2. Proposed Set of Health Ethics Principles to Guide Decision-Making in Relation to Climate Change**

**1. Stewardship and responsibility**

Authorities, financiers, the private sector, and society have a responsibility to protect and develop limited resources, and to ensure ecological integrity and human well-being. Initiatives should be implemented in a manner that most enhances human health, and the physical and social environment.

**2. Respect for persons**

Authorities, financiers, the private sector, and society have a duty to act responsibly and prudently towards each other, and towards future generations in relation to resources and in respect of initiatives that could impact on climate change and human health.

**3. Non-maleficence**

Authorities, financiers, the private sector, and society have a moral obligation not to harm, facilitate harm, or be complicit in the harm of others in relation to initiatives that could have an impact on climate change and human health.

**4. Risk-benefit analysis and burden identification**

The implications of initiatives that have an impact on climate change and human health must be timeously identified, preferably prospectively.

**5. Reasonableness and relevance**

The rationale that underpins initiatives which impact, or could impact, climate change and human health must appeal to relevant evidence, values, and principles.

**6. Collaboration**

Authorities, the private sector, the international community, and local communities should engage in collaborations to mitigate against the potential impact of climate change and adverse human health outcomes associated therewith.

**7. Least harm**

If an existing or proposed project or policy that impacts, or could impact, climate change can be realised by feasible alternatives that are less adverse to human health, these alternatives ought to be pursued as a first resort.

**8. Solidarity, duty of rescue, justice, and reciprocity**

Humans have a moral responsibility to ensure the common welfare of humankind, particularly the poor and marginalised, who are experiencing or could experience detrimental health outcomes related to climate change. This necessitates providing aid and support to these individuals.

**9. Transparency, publicity, and engagement**

The rationales and potential health implications of existing or proposed initiatives that have an impact on, or could have an impact on, climate change and human health must be publically disclosed and accessible to affected stakeholders through meaningful engagement processes.

**10. Accountability, Appeal, and enforcement**

Stakeholders who are being, or who stand to be, affected by initiatives that are impacting, or could impact, climate change and human health, must be given a fair opportunity to appeal against such initiatives, and to have their appeal upheld.

Key references: [39]–[48].

## Conclusion

Climate change represents this century's most dire environmental, food security, and public health challenge. If we are serious about negotiating a meaningful global treaty to govern climate change, the WHO, health ministers, and ethics considerations need to be at the centre of climate change policies and treaty negotiations, not at the periphery.

## Abbreviations

COP 17: 17th Conference of the Parties to the United Nations Convention on Climate Change
GHG: greenhouse gas
UNFCCC: UN Framework Convention on Climate Change
WHO: World Health Organization
